# Guidelines for the management of spontaneous preterm labor: identification of spontaneous preterm labor, diagnosis of preterm premature rupture of membranes, and preventive tools for preterm birth

**DOI:** 10.3109/14767058.2011.553694

**Published:** 2011-04-08

**Authors:** Gian Carlo Di Renzo, Lluis Cabero Roura, Fabio Facchinetti, European Association Of Perinatal Medicine-Study Group On “Preterm Birth": Aris Antsaklis, Gregor Breborowicz, Eduard Gratacos, Peter Husslein, Ronnie Lamont, Anton Mikhailov, Nuno Montenegro, Nebojsa Radunovic, Mike Robson, Stephen C Robson, Cihat Sen, Andrew Shennan, Florin Stamatian, Yves Ville

**Affiliations:** 1 Department of Obstetrics and Gynecology, University of Perugia, Perugia, Italy; 2 Department of Obstetrics and Gynecology, Hospital Vall D'Hebron, Barcelona, Spain; 3 Mother–Infant Department, School of Midwifery, University of Modena and Reggio Emilia, Italy; 4 Department of Obstetrics and Gynecology, University of Athens, Greece; 5 Department of Perinatology and Gynecology, Poznan University of Medical Sciences, Poznan, Poland; 6 Hospital Clinic-IDIBAPS, University of Barcelona, and CIBER-ER, Barcelona, Spain; 7 Department of Obstetrics and Gynecology, University of Vienna, Austria; 8 Perinatology Research Branch/NICHD/NIH/DHHS, Division of Maternal-Fetal Medicine, Department of Obstetrics and Gynecology, Wayne State University, Detroit, USA; 9 Division of Surgery, Northwick Park Institute of Medical Research, Harrow, London; 10 Department of Obstetrics and Gynecology, 1st Maternity Hospital, State University of St. Petersburg, Russia; 11 Department of Obstetrics & Gynecology, University Hospital S. João, Porto, Portugal; 12 Institute of Obstetrics and Gynecology, University of Belgrade, Serbia; 13 The National Maternity Hospital, Dublin, Ireland; 14 Institute of Cellular Medicine, University of Newcastle, UK; 15 Department ofPerinatal Medicine, Istanbul University, Istanbul, Turkey; 16 St. Thomas Hospital, Kings College London, UK; 17 Department of Obstetrics & Gynecology, University of Cluj-Napoca, Romania; 18 Department of Obstetrics & Gynecology, University Rene Descartes, Paris, France

**Keywords:** Preterm labor, premature rupture or membranes, preventive tools

## Introduction

These guidelines are based upon most recent and updated evidence and they are adapted to an European problematic by an expert view of the problem. These guidelines are not intended to be a meta-analysis or a systematic review. They follow the previous guidelines published in 2006 [1].

## Identification of preterm labor

Several biochemical and biophysical markers have been proposed for the identification of patients at risk for spontaneous preterm delivery, in both patients with threatened preterm labor and asymptomatic ones, with the hope that interventions could prevent preterm delivery [2-4]. There is now compelling evidence that examination of the cervix with ultrasound is superior to vaginal digital examination [5] and in patients presenting with preterm labor can assist in determining the risk for preterm delivery before 34 weeks. In general, the shorter the cervix, the higher the risk for preterm delivery and vice versa [2,6]. Transvaginal cervical sonography is a good method to assess the risk of preterm delivery in patients presenting with preterm labor, low-risk asymptomatic patients, and patients at high risk for preterm delivery [7,8]. Furthermore, in patients with a long cervical length (>3.0 cm), the likelihood of preterm delivery is low and, therefore, avoiding aggressive intervention in the setting of premature labor may be justified [1,2,9]. In contrast, patients who have a short cervix would have a higher rate of preterm delivery and may benefit from targeted interventions (i.e. steroid administration and transfer to a centre with a newborn special intensive care unit) [10].

A cervical length of 25 mm or less had a sensitivity, specificity, positive predictive value, and negative predictive value of 76%, 68%, 20%, and 96%, respectively, to identify preterm singleton birth at less than 34 weeks of gestation [8].

It should be also noted that endovaginal sonographic examination of the uterine cervix in women with preterm labor identifies patients at increased risk of intrauterine infections [11].

The evidence provided by several studies suggests that the assessment of the risk of preterm delivery in patients with a previous history of preterm birth or mid-trimester pregnancy loss require a longer cervix than those without such a history [6].

The assessment of the frequency of uterine contractions has been proposed to identify those at risk for preterm delivery in both asymptomatic and symptomatic pregnant patients. The rationale for this is that increased frequency of uterine contractions leads to preterm delivery. However, the results of randomized clinical trials have indicated that ambulatory uterine monitoring has not reduced the rate of preterm delivery [12].

A growing body of evidence indicates that a positive fetal fibronectin (fFN) test in cervical and/or vaginal fluids is associated with preterm delivery both in patients with threatened preterm labor and in symptomatic patients. A negative fFN test identifies patients at very low risk [4,6,13].

A positive fFN test and/or increased cytokine concentrations in cervicovaginal fluid increase the predictive value of cervical ultrasonography to identify patients at risk for preterm delivery [2,14,15].

Actim Partus (phosphorylated insulin-like growth factor binding protein 1 – pIGFBP-1) test can be used for estimating the risk of preterm delivery. The test detects pIGFBP-1 in cervical secretions. Similarly to the fFN test, the Actim Partus test has been shown to efficiently rule out the risk of preterm or imminent delivery. An advantage compared to the fFN test is that the Actim Partus test is not affected by seminal fluid, and can thus also be used on patients with recent intercourse [16]. However, the test has not been consistently associated to cervical length and scientific evidence is still lacking on its comparison with fFN data.

In a recent systematic review, it has been found that cervicovaginal fFN has limited accuracy in predicting spontaneous preterm birth in both asymptomatic and symptomatic women with multiple pregnancies because the likelihood ratios for positive and negative test results generated only minimal to moderate changes in the pretest probabilities of preterm birth. The test was most accurate in predicting spontaneous preterm birth before 32 weeks’ gestation in asymptomatic women with multiple or twin pregnancies, and spontaneous preterm birth within 7 days of testing in women with twin pregnancies and threatened preterm labor [13].

This meta-analysis suggests that only 1.6% of women with twin pregnancies and threatened preterm labor who test negative for cervicovaginal fFN will deliver in the next week. This finding could be clinically important because these women could be cared for at a primary care center rather than transferred to a tertiary care center.

However, the lack of effectiveness of clinical interventions may be due to: (1) the limitations of the current tests for the diagnosis; (2) inadequate interventions; (3) the timing of the interventions; (4) an incorrect conceptual frame work. Preterm labor is one of the great obstetrical syndromes together with small for gestational age (SGA), preeclampsia, preterm premature rupture of membranes (PROM) and fetal death [10]. Thus, preterm labor has multiple etiologies, is chronic in nature and is frequently associated with fetal disease, and the clinical manifestations in both the mother and the fetus may be adaptive in nature. Moreover, these manifestations may depend on the maternal and fetal gene-environment interaction.

## Main points

Biophysical markers, biochemical markers, or a combination of both may better identify patients at risk for preterm delivery. Ultrasonography to determine cervical length, fFN testing or a combination of both is the most useful tools in determining women at high risk for preterm labor. However, their clinical usefulness may rest primarily with their negative predictive value given the lack of proven treatment options to prevent spontaneous preterm labor (SPB). Bearing in mind the excellent negative predictive value of such tests (when fibronectin is negative and cervical length by ultrasound is >2.5 cm) we recommend that tocolytic therapy and steroid prophylaxis should be withheld.

## Diagnosis of preterm premature rupture of membrane

Approximately, 8-10% of term pregnancies will experience spontaneous PPROM prior to the onset of uterine activity. Preterm PROM-defined as PPROM, prior to 37 weeks of gestation, complicates 2-4% of all singleton and 7-20% of twin pregnancies [17-20].

A number of risk factors for spontaneous PPROM have been identified. Intra-amniotic infection and decidual hemorrhage (placental abruption) occurring remote from term, for example, may release proteases into the choriodecidual tissues and amniotic fluid, leading to rupture of membranes. Indeed, placental abruption is seen in 4-12% of pregnancies complicated by PPROM, and is more common in pregnancies complicated by PPROM prior to 28 weeks of gestation. However, whether it is the cause of PPROM or a consequence of acute uterine decompression is not known [20]. Invasive uterine procedures performed during pregnancy (such as amniocentesis, cordocentesis, chorionic villus sampling, fetoscopy, and cervical cerclage) can damage the membranes, causing them to leak, but these are rare causes of PPROM [20,21].

Rupture of the membranes typically presents as a large gush of clear vaginal fluid or as a steady trickle. The differential diagnosis includes leakage of urine (urinary incontinence); excessive vaginal discharge, such as physiologic discharge or bacterial vaginosis, and cervical mucus (show) as a sign of impending labor [21,22]. Latency refers to the interval between rupture of the membranes and the onset of labor. A number of factors are known to affect the latency period, including: gestational age, degree of oligohydramnios, sonographic myometrial thickness, number of fetuses, pregnancy complications such as intra-amniotic infection, placental abruption, or active labor [21,22].

Maternal and fetal infection is the second major complication consecutive to PPROM, as chorioamnionitis complicates 10-36% of PPROM. Early and accurate diagnosis is necessary to appropriately manage patients with PROM and to limit unnecessary intervention in patients without PROM [19]. Early and accurate diagnosis of PPROM would allow for gestational age-specific obstetric interventions designed to optimize perinatal outcome and minimize serious complications, such as cord prolapse and infectious morbidity (chorioamnionitis, neonatal sepsis) [22-25]. Conversely, a false-positive diagnosis of PPROM may lead to unnecessary obstetric intervention, including hospitalization, administration of antibiotics and corticosteroids, and even induction of labor.

Clinical diagnosis may be easy when patients are presenting with heavy watery vaginal discharge or when clear fluid can be seen leaking from the cervical os. However, recent data suggest that in 47% of the cases, clinicians are uncertain regarding the diagnosis of PPROM based on clinical examination by sterile speculum examination and patient history alone [26]. Diagnosis is indeed difficult when leakage of fluid is tiny and/or intermittent and/or ultrasound examination shows a normal to low index of amniotic fluid. In these cases, noninvasive biochemical tests can help in diagnosing PPROM.

'Classic’ tests are represented by an alkaline pH of the cervicovaginal discharge, which is typically demonstrated by seeing whether discharge turns yellow nitrazine paper to blue (nitrazine test); and/or microscopic ferning of the cervicovaginal discharge on drying. Evidence of diminished amniotic fluid volume alone cannot confirm the diagnosis, but may help to suggest it in the appropriate clinical setting [21,27,28].

Efforts to be able to confirm chorioamniotic membrane rupture with minute amounts of amniotic fluid have recently led to the development of the absorbent pad (AmnioSense). This 12 cm × 4 cm pad has a central strip that changes color with fluid with a pH >5.2 [29,30]. After contact with urine, the strip reverts to its original color when dry. This is due to the detachment of conjugate-based nitrazine molecules by the urine ammonium ions [29]. AmnioSense has undergone cytotoxicity and skin irritation and sensitization testing. The two studies of the absorbent pad currently available [29,30] suggest that a negative AmnioSense result indicates intact membranes in term and preterm gestations in 99% of cases. It remains unknown whether potential confounding substances such as semen, blood, or meconium may be distinguished from amniotic fluid by the AmnioSense pad test [31]. The effects of cervicitis, vaginitis (bacterial vaginosis), and contamination with blood, urine, semen, or antiseptic agents on traditional nitrazine or pH-based technologies has been widely documented and shown to lead to high false-positive rates [19,32-34].

The fern test refers to microscopic crystallization of amniotic fluid on drying of the vaginally collected sample. It has been shown to give false-positive results due to fingerprints or contamination with semen and cervical mucus as well as false negative results due to the use of dry swabs or contamination with blood [27,34,35]. More specifically, de Haan et al. showed false-positive and false-negative rates of 11.8% and 2.0%, respectively, for women in labor tested for amniotic fluid crystallization but for women not in labor, rates rise up to 21.2.% and 40.6%, respectively [34].

All of the abovementioned clinical methods have limitations in terms of diagnostic accuracy, cost and technical ease. Moreover, such tests become progressively less accurate when more than 1 h has elapsed after the membranes have ruptured. As such, the sensitivity and specificity for pH in diagnosing ROM ranges from 90 to 97%, and 16 to 70%, respectively, and the sensitivity and specificity for the fern test in diagnosing ROM ranges from 51 to 98% and 70 to 88%, respectively.

Because of the limitations with the current standard for the diagnosis of PPROM (namely, clinical assessment of pooling, nitrazine, and/or ferning), investigators have long been searching for an alternative and more objective test. Such tests are based primarily on the identification in the cervicovaginal discharge of one or more biochemical markers that are present in the setting of ROM, but absent in women with intact membranes. Several such markers have been studied, including α-fetoprotein (AFP), fFN, IGFBP-1, prolactin, diamine oxidase activity, β-subunit of human chorionic gonadotropin β-hCG) and placental α-microglobulin-1 in order to identify PROM [36-41]. However, results using such test have been variable (Table I). Diamine/oxidase is one of the most efficient tests with a reported sensitivity of 87.3-100% and specificity of 98-100%, but lecture based on radio-immunoassay need specific and costing equipment [22].

**Table I:** Performance of noninvasive tests to diagnose rupture of the fetal membranes.

Test/Reference	Name of test	Cutoff	Sensitivity (%)	Specificity (%)	PPV (%)	NPV (%)
Nitrazine (pH)	-	Positive/negative	90-97	16-70	63-75	80-93
Ferning and/or pooling	-	Positive/negative	51-98	70-88	84-93	87-97
AFP	ROM Check® (Adeza Biomedical Corp., Sunnyvale, CA)	>30/μg/l	90-94	95-100	94-100	91-94
Fetal fibronectin	-	> 50 ng/ml	97-98	70-97	74-93	98-100
IGBP-1	PROM-TEST® (Medix Biochimica, Kauinianen, Finland) AMNI Check® (MAST Diagnostica, Reinfield, Germany)	>3/μg/l	74-97	74-98	73-97	56-95
Prolactin	-	> 30-50/μIU/ml	70-95	76-78	72-84	75-93
Diamine oxidase		> 25 /μIU/test	83	90-100	100	89
β-hCG	-	> 40-65 /μIU/ml	68-95	70-95	73-91	78-97
Urea and creatinine	-	>0.12-0.6 mg/dl	90-100	87-100	94-100	91-100
AmnioSense Absorbent pad		pH > 5.2	98.3	70	65-70	98
Lactate	Lac test®	≥4.5 mmol/1	79-86	88-92	88-92	78-87
PAMG-1	AmniSure® Test ROM (AmniSure® International LLC, Cambridge, MA)	>5.0 ng/ml	98-99	88-100	98-100	91-99

AFP, alpha-fetoprotein; β-hCG, beta-subunit of human chorionic gonadotropin; IGFBP-1, insulin-like growth factor binding protein 1; NPV, negative predictive value; PAMG-1, placental alpha-microglobulin 1; PPV, positive predictive value (modified from reference [22] and [31]).

To reduce false-positive rate, the test should identify a protein present in high quantity in amniotic fluid compared with other physiological fluid such as maternal blood, vaginal secretion, and seminal fluid.

IGFPB-1 and placental alpha microglobulin-1 (PAMG-1) are fulfilling these criteria and can be detected with respectively the Actim Prom™ test and the most recently developed Amnisure® ROM test [38,39,42,43].

IGFBP-1 is a 28 kDa protein produced by fetal liver and decidua. The IGFBP-1 protein is present in amniotic fluid in large concentrations, but absent from seminal plasma, urine, and maternal blood [42]. Concentration in amniotic fluid increases with gestational age from 27 ng/ml in early pregnancy to 145,000 ng/ml at term, whereas maternal blood concentration varies between 58 and 600 ng/ml. Actim Prom™ Test (Medix Biochemica, Kauniainen, Finland) has a lower detection limit of 25 ng/ml. The result is either positive (IGFBP-1 is present; threshold exceed 30 μg/l), or negative (IGFBP-1 less than 30 μg/l) obtained within 10-15 min of performing the test. Its sensitivity varies from 74 to 100% and specificity from 77 to 98.2% [38,42,44-46]. So the test is specific to amniotic fluid and sensitive enough to help to diagnose also micro ruptures. This test has been in wide clinical use for over a decade.

PAMG-1 is a 34 kDa glycoprotein synthesized by the decidua. Amniotic fluid concentration ranges from 2000 to 25000 ng/ml and maternal blood concentration from 0.5 to 2 ng/ml.

Amnisure® ROM Test (AmniSure® International LLC, Boston, MA) has a lower detection limit of 5 ng/ml with a sensitivity close to 99% and specificity varying between 87.5 and 100% [39,43,47]. Moreover, some investigators have proposed that concentrations of PAMG-1 in cervicovaginal fluid in patients without clinical proof of ROM may represent evidence of microleakage of amniotic fluid. Amnisure ROM test was performed in patients without evidence of clinical ROM. Patients in labor without clinical ROM, but with a positive Amnisure test had a significantly shorter admission-to-delivery interval than patients in labor without clinical ROM with a negative Amnisure ROM test [47,48]. Lee et al. demonstrated that the Amnisure test has a better diagnostic accuracy than combined use of nitrazine, fern and pooling, as well as the nitrazine test alone.

As amniotic fluid sample collected in the vagina is systematically contaminated with vaginal discharge, the detection limit is an important parameter to consider for the performance of the test. The detection limit of PAMG-1 with Amnisure ROM test (5 ng/ml) is lower than the limit detection of IGFBP-1 with Actim PROM test (25 ng/ml). [22,38,49]. Recent investigations into the effect of high blood admixture to the patient sample on the PAMG-1 test have shown that blood admixtures as high as 50% do not interfere with the PAMG-1 test [50]. Semen and urine do not interfere with PAMG-1 test either, as both substances do not contain PAMG-1 protein.

Lack of noninvasive gold standard test for PPROM is a severe limitation for investigating new diagnostic tests. Ideally, a gold standard test would be an amnio-dye test consisting of amniocentesis for instillation of indigo carmine in amniotic cavity and research for leakage of blue-stained fluid into the vagina within 20-30 min [51]. This method, however, presents the disadvantage of being invasive and carries risks of ROM and infectious complications. Only PAMG-1 test has been compared to amnio-dye test. Preliminary results of this study were published recently and indicate that PAMG-1 test is as reliable as amnio-dye test in diagnosing ROM [51].

A limited number of quality studies and the limited number of cases with preterm birth per study seriously constrain the conclusions regarding the reliability of different ROM diagnostic methods. As spontaneous preterm birth has low prevalence, particularly for important outcomes such as birth before 34 weeks’ gestation or birth within 48 h of presentation, the small absolute numbers of affected cases introduced imprecision by increasing variance. All that said, the relative performances of the diagnostic tests used in the various studies, regardless of the gold standard to which they were compared, have continuously suggested that the test based on PAMG-1 detection is more reliable and noninvasive than other methodologies [26,38,39,43,52].

## Main points

1. PPROM complicates 2-20% of all deliveries and it is associated with 18-20% of perinatal deaths. Management options include admission to the hospital and administration of antenatal corticosteroids. Amniocentesis to exclude intra-amniotic infection and/or broad-spectrum antibiotics prophylaxis are further options.
2. The clinical signs of PPROM documented on sterile speculum examination are copious isualpooling of fluid in the vagina or leakage of fluid from the cervical os. Complementary evidence includes an alkaline pH of cervicovaginal discharge, and/or microscoping ferning of the cervicovaginal discharge on drying.
3. The clinical signs of PPROM become progressively less accurate when more than 1 h has elapsed after membrane rupture. Evaluation of ferning, nitrazine, and/or ultrasound has shown that they add little, if anything, to speculum examination alone and that none of them are as accurate as the test based on biochemical markers. Accordingly, we believe that there is little to merit their use in modern practice.
4. Investigators search for a test based primarily on the identification in the cervicovaginal discharge of 1 or more biochemical markers that are present with ROM, but absent in women with intact membranes [22,30]. Biochemical markers are better than the traditional methods, as they are specific to proteins found in amniotic fluid. Thus they are not affected by most contaminating substances and enable a fast and reliable bedside diagnosis.
5. PAMG-1 test is most useful tool in determining women at high risk for premature rupture of fetal membranes. The rapid strip test based on PAMG-1 seems to be the more accurate bedside test compared with others [26,38,49, 51,52].

## Preventive tools

### Cervical cerclage

The use of cervical cerclage has been one of the preventive strategies used for many years; however, there are no studies that show overall evidence except in very specific cases [53-55]. It is clear that the use of cerclage based on a short cervix has any effect on prevention of prematurity [54,56]. The literature shows evidence that cerclage provides clear and proven benefits only in circumstances diagnosed with ‘cervical incompetence'. In cases of a previous history of three or more late abortions, or three or more preterm delivery, cerclage performed in the first half of pregnancy in patients with a single fetus shows a statistically significant beneficial effect [57-59]. Cerclage may have a beneficial effect in preventing preterm delivery when there is a history of preterm labor and an objective decrease in cervical length or increase cervix dilatation in non-symptomatic patients [55,60-62]. In cases with uterine abnormalities, cerclage has failed to show evidence of improvement in perinatal results [58]. Also, in cases of twin pregnancies cerclage has even shown a deleterious effect (increasing paradoxically the rate of preterm delivery), therefore it is not recommended in these settings [55,61,63]; nor it has been proved effective in cases with previous cervical conization [64]. In cases with advanced cervical dilation and uterine contractions, the use of emergency cerclage associated with the administration of tocolytic agents has shown controversial effects [55,60,65,66]. There are no differences between the Shirodkar or MacDonald type of cerclage [67,68]. The cerclage should be performed in absence of contraindications such as placenta previa, cervical or vaginal infections, amniotic infection, uterine bleeding, fetal malformations, fetal death or distress or alterations of the amount of amniotic fluid (polyhydramnios or oligohydramnios), PROM, or maternal contraindications [69]. The most common complication of cerclage is PROM and amniotic infection and therefore appropriate controls of infections should be carried out [70]. Likewise, at the time of delivery, the presence of a previous cerclage has been associated to increased cervical dystocia. It is important to observe that a marker of appropriate placement of the cerclage is the distance from cerclage and the internal cervical os, as measured by ultrasound. A measure of 10 mm represents a good surgical result [71,72].

### Cervical pessary

Many years ago, the cervical pessary was used for cervical incompetence with very inconsistent results [73]. In recent years, it has been considered the preventive effect on preterm delivery of placing a cervical pessary in non-symptomatic patients, with singleton pregnancy and a short cervix (less than 25 mm, at 20-24 weeks gestation as risk marker), without prior cervical incompetence [74,75]. Various studies show significant risk reduction without increasing the rate of vaginal infections [76,77]. Only a properly designed, prospective, randomized study has confirmed these results (28 *vs* 5%), so this preventive strategy must be analyzed with caution [76,77]. Similar studies in patients with twins are being conducted, with no conclusive results. Therefore, these guidelines can only be intended as source of information of this possibility and recommended for use only in research protocols.

### Progestogens use in pregnancy

The knowledge that an increased activity of endogenous progesterone (P4) was a necessary event for the development and the maintenance of pregnancy dates back to the first half of the last century [78]. Around the 60s we acquired the idea that a withdrawal of endogenous P4 was related to the onset of labor [79] even preterm [80]. Since then, P4 and related synthetic compounds such as 17 α-hydroxy progesterone caproate (17 OHP-C) as well as other progestogens have been tested in clinical trials to prevent the challenging phenomenon of preterm birth (PTB).

In one of the first meta-analyses ever published about perinatal interventions, it was demonstrated that 17 OHP-C treatment was associated with a reduced rate of PTB (both preterm delivery less than 37 weeks and babies weighting less than 2500 g) in respect with placebo or no intervention [81]. Surprisingly, such achievement was not implemented into clinical practice, nor scientific societies endorsed such conclusions producing recommendations. The William's Obstetrics textbook 21st edition released in 2001 did not mention progesterone among interventions able to prevent PTB and stated (p. 270): ‘Progesterone administration to pregnant women does not… arrest or prevent preterm labor’ [82]. Clinical and experimental studies re-started in the years 2000 [83]. Now, an almost equal number of randomised controlled trials (RCTs) and meta-analyses is available while several trials are still being planned or are ongoing.

The vast majority of such clinical trials were performed with diverse formulations of either P4 or 17 OHP-C [84]. P4 has been administered through daily vaginal route by using two different pharmaceutical preparations, i.e. 8% gel or 100-400 mg micronized hormone. On the other hand, 17 OHP-C has been administered through intramuscular injection, by using doses ranging 250-682 mg/week, with drug dissolved in castor or ethyl oil. Given the different biological actions of P4 and 17 OHP-C [85] and considering that we still ignore the mechanism(s) of action of such treatments it seems difficult at present to put together the results of all these RCTs under the umbrella of ‘progesterone treatment’ [86].

A further source of heterogeneity which refrains from summarizing published data into guidelines is represented by the inclusion criteria utilized in the different studies [87]. The most part of randomized subjects is represented by women with a history of at least one previous spontaneous PTB or by multiple pregnancies. However, asymptomatic mid-second trimester women with a very short cervix as well as third-trimester patients having had a successful treatment of a preterm labor episode were also admitted to ‘progesterone’ supplementation.

Micronized progesterone capsules (200 mg vaginally daily) were used in the trial of P4 for asymptomatic women with a very short cervix (less than 15 mm), and appeared to be effective for such an indication [6]. Whether the differences seen in efficacy of the recently studied vaginal preparations reflects differences in dosages (100 mg *versus* 200 mg), variation in absorption and bioavailability with different preparations (gel *versus* capsule versus suppository), or differences in study populations remain to be elucidated [88]. Supplemental 17 OHPC treatment does not benefit women with short cervix and previous preterm birth submitted to cervical cerclage for suspected cervical insufficiency. Interestingly, if the same women did not receive a cerclage, 17 OHPC reduced perinatal mortality [89].

Neither progesterone nor 17 OHPC has been studied as a preventive agent for asymptomatic women with a positive cervicovaginal fFN screen result or as therapeutic agent in PPROM. Both P4 and 17 OHPC has been proven effective in the tertiary prophylaxis of preterm birth after tocolysis [86,90].

Concerning safety issues pertaining the prolonged use of P4 or OHP-C, neither progesterone nor 17-OHP-C consistently adversely affected maternal weight, embryo-fetal viability, or caused malformations in non-clinical studies conducted in mice, rats, rabbits, guinea pigs, horses, or non-human primates. There is a signal for embryo-fetal toxicity associated with 17-OHP-C in the two largest clinical trials conducted to date; there is also a signal for embryo-fetal toxicity with 17-OHP-C in rhesus monkeys and possibly one in rodent species. The relationship between these signals is unclear given the absence of state-of-the-art reproductive toxicology studies and human pharmacokinetic studies [91]. The effects of 17-OHP-C upon pregnancy in experimental animals have been studied in rats, rabbits, mice, and monkeys. Earlier studies found no evidence of androgenic or glucorti-coid activity and no virilising effects on female fetuses [92-96]. It is worth noting that the synthetic 17-OHP-C and natural progesterone are not similar molecules and have different activities in a number of respects including their effects on the myometrium [85,97-100]. Natural progesterone has documented properties of inhibiting uterine contractions [85,98,100], whereas 17-OHP-C seems to have no effect on uterine contractions [85,99]. In addition, natural progesterone has an established safety profile in the first trimester of pregnancy from more than 11 years of continued and ongoing use in infertility as daily progesterone supplementation and replacement in IVF cycles [101]. Furthermore, in a recent very large preterm birth prevention study of singleton pregnancies, no cases of miscarriage associated with the use of micronized natural progesterone were observed [102]. On the other hand, 17-OHP-C is associated with an increase in resorption (miscarriage) in pregnant rats [96], total embryo-lethality in pregnant rhesus monkeys [103], a signal for a 30% increase in miscarriage in a meta-analysis of 17-OHP-C clinical studies [81], as well as an imbalance in miscarriage associated with 17-OHP-C in the largest placebo controlled randomized trial published to date [83]. In a study by Rebarber et al. [104], patients who received prophylactic treatment with 17-OHP-C had a higher incidence of gestational diabetes (odds ratio 2.9 [95% CI: 2.1-4.1]) than those who were not treated. The latter study suggests that treatment with 17-OHP-C may be associated also with increased maternal morbidity that is an additional safety flag.

## Main points

Considering all of the above reported limitations and based on the primary studies published to date, the following statements could be actually advised.

1. In asymptomatic women presenting with prior history of PTB, the early prophylaxis with either P4 microionized or 17 OHP-C demonstrated to be efficacious in preventing recurrence [83,98,102,105-108]. In the above reported conditions, we advice to implement prophylaxis (200 mg vaginal P4 or 250 mg/weekly i.m. 17 OHP-C) since early second trimester, in such condition.
2. In single pregnant, nulliparous women where a silent cervical shortening (15 mm) could be detected with transvaginal ultrasound both microionized P4 and 17 OHP-C have proven to be able to reduce PTB, in respect with placebo [6,89,109]. Two good quality studies performed in few subjects support this intervention which, however, requires further confirmation before being recommended in the clinical practice.
3. In single pregnant, nulliparous women successfully treated for a preterm labor episode microionized P4 reduced the rate of PTB in respect with no intervention/placebo [90,110]. The use of progestogens (400 mg/daily vaginal microionized P4 or 375 mg/twice a week i.m. 17 OHP-C) as a maintenance tocolysis, however, requires further studies before being recommended for the tertiary prophylaxis of PTB.
4. In multiple pregnancies, either twins or triplets, neither microionized P4 nor 17 OHP-C is able to prevent PTB [111-114]. Data are consistent and number of women studied enough to advice not to use progestogens in such condition [115].
5. Maternal safety of either microionised P4 or 17 OHP-C administration has been reported in different trials [97]. Neonatal safety has been evaluated in only one trial where mothers have been treated with 17 OHP-C [116]. No effects of general health status, external genitalia, and psychomotor development have been reported at follow-up. However, there is concern about the increase in fetal death in mid-trimester and the higher incidence of gestational diabetes linked to 17-OHP-C. Since the paucity of data, ongoing trials are encouraged to include neonates follow-up in their design. Moreover, in view of the widespread use of progestogens in pregnant women, physicians should be aware of these facts for proper informed recommendation about the use of 17-OHP-C and post-marketing surveillance has to be advised.
